# Stronger declines in youth alcohol consumption thanks to stronger integrated alcohol policies? A qualitative comparison of ten Dutch municipalities

**DOI:** 10.1186/s13011-017-0091-8

**Published:** 2017-03-02

**Authors:** Moniek C. M. de Goeij, Janneke Harting, Anton E. Kunst

**Affiliations:** Department of Public Health, Academic Medical Center (AMC) - University of Amsterdam, PO Box 22660, Amsterdam, 1100 DD The Netherlands

**Keywords:** Integrated alcohol policies, Integrated public health policies, Alcohol consumption, Youth, Cross-sectoral collaboration, Media, Intervention strategies

## Abstract

**Background:**

Little detailed evidence is available on how integrated policies could impact population health and under what conditions such policies could be realized. The aim of this study was to assess how youth alcohol consumption trends in the province of Noord-Brabant, The Netherlands, were related to the development and implementation of integrated policies.

**Methods:**

In a retrospective multiple case study, alcohol policies of six municipalities with stronger declines in youth alcohol consumption between 2007 and 2011 (cases) were compared to four municipalities with weaker declines (controls). Information on the policy process in the same period was obtained through semi-structured in-depth interviews with policy advisors. Information on implemented interventions was extracted from policy documents and checked by the interviewees. Interviews were analyzed for thematic content.

**Results:**

Only municipalities with stronger declines in alcohol consumption involved sectors other than public health and had started to implement interventions that use regulatory or enforcement strategies. Their involvement was facilitated by framing youth alcohol consumption as a safety rather than a health problem, whereby local media played a substantial role. Implementation of integrated policies was further facilitated by dedicated leadership and sufficient resources.

**Conclusions:**

Reductions in youth alcohol consumption in Noord-Brabant were stronger when municipalities started to develop integrated policies. Results suggest that integrated policies framing a health problem as a broader societal problem could positively influence population health.

## Background

Integrated public health policies [1–5] are policies developed by multiple policy sectors in order to implement multiple intervention strategies that target a variety of determinants of health. This type of policy is recommended to improve health-related behaviours, such as smoking, drinking, and obesity, because these behaviours are influenced by both personal (e.g., knowledge, and attitude) and environmental determinants (e.g., physical, and social environment) [6–8]. In addition to educational strategies, that mainly address personal determinants, the package of implemented interventions should include other strategies, such as facilitation and regulation, to address the environmental determinants [2, 9]. To achieve such a variety in used intervention strategies, it is suggested that the public health sector should jointly develop and implement policies with other sectors (e.g., safety, and education) [1]. Little evidence is available on how integrated policies should manifest themselves to exert an effect on health-related behaviours and how such effective policies can be realized.

Many studies have identified factors that are important for the development [9–11] and implementation [12–15] of integrated public health policies, and also specifically for cross-sectoral collaboration [16, 17]. Examples of such factors are priority, support, management or leadership, awareness, motivation, sufficient resources (e.g., time, and money), knowledge, skills, and interventions’ sustainability. However, potential influential factors have scarcely been linked to the actual output of integrated policies (e.g., implementation of varied interventions to address environmental determinants of health) and the subsequent impact on health-related outcomes (e.g., improvement in health-related behaviours, or physical health). To our knowledge, while one study investigated the impact on subjective health measures [18] evidence from studies using objectively measured health-related outcomes are lacking. More knowledge on the complete policy process is essential for scientists and policy makers to identify those factors that make integrated policies not only feasible, but also effective in realising output and improving population health. Knowledge about effects on health-related outcomes can also be used by policy makers to either justify their choice for implementing integrated policies or to perform cost-benefit analyses.

To investigate associations between factors of integrated policies and health-related outcomes, the Dutch province of Noord-Brabant was a useful case as both measures varied across regions and municipalities. Between 2007 and 2011, three regional alcohol prevention projects were running, and within these projects, municipalities had control over what they implemented locally. In an earlier study conducted in 56 municipalities in Noord-Brabant, we found that municipalities with policy documents formulating stronger integrated alcohol policies, in terms of more varied intended interventions, had more favourable trends in youth alcohol consumption than municipalities with weaker integrated alcohol policies [19]. However, with the collected policy documents we were not able to identify the interventions that had been actually implemented and to identify factors related to the development and implementation of such interventions. Therefore, our aim was to assess in more detail how youth alcohol consumption trends were related to the development and implementation of integrated alcohol policies. We do so by comparing alcohol policies between two groups of municipalities in the Dutch province of Noord-Brabant with contrasting trends in youth alcohol consumption.

## Methods

### Design

We used a retrospective multiple case study, in which we combined an analysis of policy documents with in-depth interviews with policy advisors (i.e., policy officials, and health promotors) of six municipalities with ‘stronger’ and four with ‘weaker’ declines in youth alcohol consumption.

### Selection of municipalities

The first step was to select the 25% largest municipalities in the Dutch province of Noord-Brabant (*n* = 17) based on the number of inhabitants in 2007 (≥36,645 inhabitants). This selection made it more likely that adolescents had been actually exposed to the implemented integrated alcohol policies in that municipality, as the presence of secondary schools, sports clubs, and bars lowers the chance that they migrate to other areas for these facilities.

The second step was to estimate the trend – defined by the change in prevalence between 2007 and 2011 – in six alcohol measures among adolescents aged 12 to 18, for each of the 17 municipalities. These trends were estimated with data obtained from the Youth Health Monitor 2007 and 2011, which is a 4-year survey implemented in the three regional Public Health Services in Noord-Brabant [19]. The following alcohol outcome measures were defined: mean age on which adolescents consumed their first glass of alcohol (age of onset – truncated at age 8), and the prevalence of ever drinking (≥1 drinks during their life), regular drinking (≥1 drinking occasions during the last 4 weeks), weekly drinking (≥0.5 glasses per week), heavy episodic drinking (≥5 glasses on one occasion, ≥1 times during the last 4 weeks), and heavy weekly drinking (≥5 glasses per week, among the weekly drinkers). Trends in youth alcohol consumption were analysed with a linear regression analysis (with mean age of onset as outcome) or logistic regression analysis (with either ever, regular, weekly, heavy episodic, or heavy weekly drinking as outcome) in R version 3.2.0 statistical software. Time (i.e., 2007, and 2011) was included as an independent variable in the model and sex, age, education, and ethnicity were included as control variables. For the measurement of these control variables we refer to our previously published study [19].

The third step was to select municipalities with contrasting trends in youth alcohol consumption. This selection was based on a composite rank score for each municipality that adds up the rankings of that municipality on each of the six alcohol outcome measures. For each alcohol measure, the ranking of that municipality was obtained by ranking all 17 municipalities according to their beta from the regression model. A more negative (more positive for age of onset) beta represents a more beneficial change in the prevalence of this alcohol measure between 2007 and 2011. We selected four of the municipalities with the lowest composite rank scores. These municipalities were individually matched to a municipality out of those with the highest scores. This matching was based on two factors that can possibly confound the development and implementation of alcohol policies: 1) the absolute level of youth alcohol consumption in 2007, and 2) the number of inhabitants in 2007. The size of a municipality – based on the number of inhabitants – influences relevant factors such as the presence of secondary schools and the number of bars and clubs, and thereby the type of interventions that may need to be implemented. This matching resulted in four pairs and thus eight municipalities. In addition to these four pairs, we also selected the two remaining unmatched large municipalities – which both had stronger beneficial trends in youth alcohol consumption – because we anticipate that interviews with policy advisors of larger municipalities give more extensive and detailed information. Table 1 describes the composite rank score and individual rankings of all six alcohol outcome measures for each of the ten selected municipalities.

**Table 1:** Selection of municipalities

Municipality	Alcohol measure ranking						
Name	Mean age of onset	Ever drinking	Regular drinking	Weekly drinking	Heavy episodic drinking	Heavy weekly drinking	Total ranking
Four municipalities with weaker declines in youth alcohol consumption							
1W	5	1	4	5	2	2	19
2W	6	2	3	2	3	8	24
3W	2	6	5	3	8	14	38
4W	11	13	2	4	9	9	48
Six municipalities with stronger declines in youth alcohol consumption							
5S	1	17	13	16	7	5	59
6S	12	5	11	12	13	11	64
7S	13	8	14	15	11	3	64
8S	14	11	10	8	12	12	67
9S	10	14	6	9	14	15	68
10S	16	16	8	13	16	17	86

**Fig. 1:**
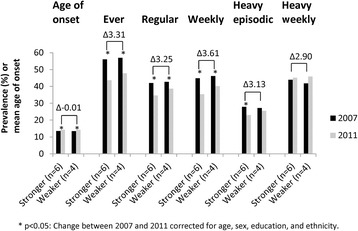
Trends in youth alcohol consumption

The fourth step was to assess to what extent these ten municipalities indeed had contrasting trends in youth alcohol consumption. Figure 1 shows that between 2007 and 2011, the prevalence of ever, regular, weekly, and heavy episodic drinking decreased with an additional 3.31, 3.25, 3.61, and 3.13%, respectively, in the six municipalities with stronger versus the four municipalities with weaker declines in youth alcohol consumption. The age of onset increased similarly in both groups of municipalities, while the prevalence of heavy weekly drinking remained stable in both groups.

### Alcohol policies

The following phase in our study was to qualitatively assess, between 2007 and 2011, the composition of the package of implemented interventions, the foregoing policy process, and the factors playing a role in this process.

To assess the composition of the package of implemented interventions, for each municipality, a summary of the policy goals and intended alcohol interventions (describing the implementer, type of intervention strategy, target group, and setting) was made. The summary was based on policy documents from five sectors (i.e., Public Health, Youth, Safety, Events, and Catering Industry) collected in our previous study [19].

To assess the foregoing policy process and influential factors in each municipality we used semi-structured in-depth interviews with open-ended questions (Table 2). Questions were based on a topic list developed by all authors. Included topics covered input (i.e., resources, such as time and money), policy development process (i.e., reasons to develop policy, collaboration between and role of different sectors, priority, possible factors influencing development), and policy output (i.e., implementation of interventions, possible factors influencing implementation). This list was based on insights from epidemiological publications on public health, publications on integrated public health policies, and conceptual notions and empirical findings from policy and implementation sciences [18]. An additional question was added at the beginning of the interview (Table 2) to check whether interventions formulated in the policy documents were actually implemented or whether there were interventions missing.

**Table 2:** Open-ended questions and coding list

*Questions*	
*How effective integrated alcohol policies should manifest themselves*	
1. In preparation of this interview, I sent you a document containing a summary of the intended alcohol policies in municipality X (2007–2011). Are these interventions actually implemented? Are there interventions missing? Can you supplement?	
2. What was the reason for municipality X to decide whether or not to focus on alcohol policies?	
3. Why do you think that municipality X chose this package of alcohol interventions?	
*How effective integrated alcohol policies can be realized and which influential factors play a role*	
4. Who was the initiator of alcohol policies in municipality X?	
5. What was the support for alcohol policies within municipality X?	
6. What (specific or additional) resources did municipality X have when developing alcohol policies?	
7. Did municipality X collaborate with other sectors than Public Health when developing alcohol policies? If yes, with whom and what was the role of these partners?	
8. What was the specific role of the regional Public Health Service when developing alcohol policies in municipality X?	
9. What are in your opinion important barriers and facilitators when implementing alcohol policies? Can you give examples from your own experiences?	
10. What other things happened in municipality X, next to the discussed alcohol policies, that could have influenced changes in youth alcohol consumption?	
*Main codes*	*Sub codes*
Initiation	Start of development process
Collaboration	Regional alcohol prevention project
	Local working group
Development	Alcohol policies
	Policy document
Public Health Service	Leading role
Municipal board	Politics
	Council
	Mayor
	Alderman
	Civil servant
Resources	Money
	Time
Involved actors	Policy sectors
	External actors
Context	
Alcohol intervention plan	
Media	
Alcohol interventions	Target group
	Setting
	Determinants

### Data collection

In April 2014, we approached the regional Public Health Service employees who advised the ten selected municipalities on alcohol policies. One person was interviewed twice as this person advised two different municipalities. For two municipalities, both with stronger declines, the same four persons were interviewed concurrently as they all played a role in both municipalities. One of those four persons was also interviewed for the third municipality included from that region. In total, ten advisors were interviewed.

Interviews were conducted at regional Public Health Services and lasted approximately 45–60 min. The first author conducted, audio-taped, and anonymized the interviews, while a student assistant fully transcribed them. At the interview start, policy advisors were unaware whether their municipality had stronger or weaker declines in youth alcohol consumption.

### Analyses

Interviews were analysed for thematic content with the software program MAXQDA 11. The first two authors extensively read and open coded five interviews, which was partly guided by the themes in our topic list. Consensus on this list was reached through extensive discussions between the two. Thereafter, both authors again coded the same five interviews by using the preliminary coding list. After additional discussions the coding list was extended and refined resulting in consensus on which main and sub codes to use. The final coding list contained both themes from our topic list as well as a few new emerging themes (Table 2).

After all interviews were selectively coded by the first author, information on the implemented interventions and on the coded themes was tabulated for each municipality. Thereafter, municipalities were ordered based on the strength of decline in youth alcohol consumption (Table 1). With this tabulation we could perform constant comparison of all municipalities to recognize patterns within the relations between effects on youth alcohol consumption, implemented alcohol interventions, and the foregoing policy process. Main results were discussed with all authors. To support the results, we report quotes from policy advisors of the four municipalities with weaker (Table 1, 1W to 4W) and six municipalities with stronger (Table 1, 5S to 10S) declines in youth alcohol consumption.

## Results

### Package of alcohol interventions

Table 3 gives an overview of the type of policies identified amongst the municipalities with, respectively, weaker or stronger declines in youth alcohol consumption. More specifically, the table shows the intervention strategies used, population subgroups targeted, and settings in which interventions were implemented. Municipalities with stronger declines appeared to have implemented a package of more varied interventions than municipalities with weaker declines. First, these municipalities implemented interventions with educational strategies in multiple settings (e.g., schools, sports clubs, and bars) instead of merely in a school setting. Second, these educational interventions not only targeted adolescents but also their parents. Third, some municipalities started to develop regulatory and enforcement strategies, including enforcement of local sales regulations by the Drug and Food administration and care trajectories for juvenile delinquents being arrested when drunk. Some of the strategies were developed or implemented regionally. Their development was however at an initial stage.

**Table 3:** Intervention strategies, target groups and settings identified in the municipalities with, respectively, weaker or stronger declines in youth alcohol consumption

	Municipalities with weaker declines	Municipalities with stronger declines
Educational strategies	*Target group*: Adolescents *Setting*: At primary and secondary schools	*Target group*: Adolescents; Parents *Setting*: At primary and secondary schools; At sports clubs; During events; On the street; In bars
Non-educational strategies		*Regulation*: Writing of regulatory policy *Enforcement*: Mystery shoppers in sports clubs, bars, and supermarkets from the regional alcohol prevention project; Enforcement by the Drug and Food administration initiated from the regional alcohol prevention project; Juvenile delinquent trajectories after being arrested when drunk
Media strategies		*Negative media attention *(for example in newspaper) about negative consequences of Happy Hours; Levels of alcohol consumption; Offenses of regulations *Positive media attention *about covenants with sports clubs or bars

*A: “Yes, you will see this in what has been achieved. Those are all educational activities.” Q: “But in different contexts?” A: “Yes, in different contexts and they were of course working on writing regulatory policies and such, they have been doing that.” Q: “Yes, so certain things were in a developing phase?” A: “Yes, absolutely. But really enforcement, no … that is not performed.”* [8S]

A new emerging intervention strategy was the use of media, as three municipalities with stronger declines participated in the same regional alcohol prevention project that often used media channels to communicate alcohol interventions to the public to increase public awareness on and uptake of interventions.

*A: “And then there was a club [sports club] that closed a covenant [on regulations], which was broadly reported in the media.”* [8S]

*A: “But it was the case that the newspaper reported violations [of regulations] found somewhere in the municipality.”* [7S]

### Indicated reasons to implement alcohol interventions

In municipalities with stronger declines, advisors indicated that interventions with enforcement/regulatory strategies could be developed and implemented because of incidents of drinking-related youth nuisance and the wide media attention it received.

*A: “Well that was picked up more from the nuisance enforcement.” Q: “Exactly, because of youth nuisance in nightlife areas.” A: “Yes.”* [10S]

*A: “… but that it [number of underage adolescents that got drinks in alcohol outlets] was reported in the newspaper, that was interesting.” Q: “Media attention, yes.” A: “And then, then municipalities would also say: ‘And how many in our municipality?' And they did not hear this, but it has been very much agenda-setting, I think, and awareness.”* [7S and 9S]

*A: “… it was that alcohol train which came broadly in the news, because it was again demolished…”* [8S]

High levels of youth alcohol consumption did not play a decisive role in most municipalities with stronger declines, but in some it played an additional role.

*A: “[No] We [municipality] are just performing the best [regarding youth alcohol consumption] of the entire region … and we [municipality] have other problems.”* [9S]

*A: “[Yes] Well, I think mainly the numbers [on youth alcohol consumption] from the youth health monitor.”* [5S]

In municipalities with weaker declines neither youth nuisance nor high levels of youth alcohol consumption gave rise to the development or actual implementation of interventions including enforcement or regulatory strategies. Apart from experiencing other health-related problems, these municipalities perceived levels of alcohol consumption as lower than in surrounding municipalities, but this perception could not always be supported by measured levels.

*A: “As far as I can recall, the municipality has not scored very negative compared to the region, but is it more betting on national priorities.”* [2W]

### Collaboration between different sectors

#### Collaboration at the regional level

The package of varied interventions implemented in municipalities with stronger declines was primarily initiated from a regional project that started to develop an integrated approach before 2007. In response to problems of youth nuisance, policy advisors reported the formation of three regional working groups (i.e., education, regulation, and enforcement) that included actors, both inside and outside the municipal organization, from other sectors than public health. The regional Public Health and Addiction Service were the predominate public health actors involved in the development and implementation of alcohol policies. According to the policy advisors, non-public health actors could be involved during policy development, such as the Drug and Food Administration that helped to develop enforcement policies, or during policy implementation, such as the Juvenile Delinquent Service that implemented educational trajectories for adolescents that broke the law (e.g., drinking in public areas).

*A: “[Inside] Because they [the regional project] had those three working groups, they [the regional project] also had people in the working group regulations, not from the regional Public Health Service so to speak, but from municipalities that had something to do with regulations.”* [7S]

*Q: “[Outside] And here [written document] it is stated: for further elaboration and implementation of action plans, key partners, such as police, prosecution office, Food and Drug Administration, municipal welfare, and the council working group ‘Catering industry’ policy plan, were explicitly involved.” A: “Exactly.”* [8S]

In contrast, in municipalities with weaker declines where drinking-related youth nuisance did not play a prominent role, it appeared to be difficult to involve actors, both inside and outside the municipal organization, from other sectors than public health.

*A: “It was difficult to … to really … I think to get that on the agenda of … of the ‘harder’ side [*i.e.*, sectors other than public health] let’s say within the municipality. The ‘harder’ sectors …”* [4W]

#### Collaboration at the municipal level

Collaboration between public health and other sectors inside the municipal organization, such as enforcement and safety, was indicated to be limited in all municipalities. Involvement of different sectors in local working groups was present in a few municipalities with stronger declines, but knowing about policy goals and implemented interventions across sectors seemed to be absent.

*A: “… well, maybe communication, but no collaboration.”* [10S]

* A: “… especially at the authorization and enforcement side internally in the municipality. That the link was not judged to be logical … that it [authority and enforcement] would have to do something with health: Why? Very much that compartmentalised actually, that has changed but was present then.”* [4W]

In addition, in the majority of municipalities, no working groups were initiated at the municipal level. Only two municipalities with stronger declines had a local working group that involved actors, both inside (e.g., enforcement, and safety) and outside the municipal organization (e.g., police, and the regional Addiction Service), from other sectors than public health.

*A: “… and they also wanted to reduce youth nuisance, and from there, so actually not from public health but from enforcement and safety, that local working group originated and the local working group was integral.”* [6S]

### Leadership

The policy advisors indicated that in none of the municipalities with weaker declines the municipal board took up political leadership.

*Q: “And, let’s see. Yes, the collaboration with other policy sectors was hardly present. And with external actors and policy implementers it was present during development but the step thereafter did not take place?” A: “Yes, that did not take place, no. I think this has nothing to do with bad collaboration relations, but purely with the leading role from the municipality.”* [3W]

In the majority of municipalities with stronger declines, it seemed that no leading role was yet taken in the period 2007–2011.

*A: “And there were a few recommendations from which the municipality could pick out what went wrong. That was, amongst other things, that the leading role was not taken enough…”* [9S]

The absent political leadership of the municipal board might be explained by their believe that enough was being initiated within the regional alcohol prevention project in which they participated.

*A1: “Because they [aldermen from other municipalities] were very active and this municipality actually did nothing. They went along with the regional alcohol prevention project.” A2: “Many municipalities thought: ‘Well, we participate in the regional alcohol prevention project, so we have alcohol prevention, that is arranged’.”* [7S]

The municipal board seemed to however take a leading role in two municipalities with stronger declines.

*Q: “And why did that [local working group/integrated alcohol policy] succeed in this municipality?” A: “They [municipality] initiated this [local working group/integrated alcohol policy] themselves, you see. We [regional Public Health Service] did not have to push them [municipality].”* [8S]

In the majority of municipalities with stronger declines and lacking political leadership of the municipal board, policy advisors reported that the regional Public Health Service showed strong program leadership. They mentioned that this Service took the initiative to initiate interventions and to stimulate municipalities to take some action themselves.

*A: “It [implementation of interventions] really comes from us and that is also the case for all other interventions.”* [5S]

*A: “… but those staff [of the regional Public Health Service] were also trying to stimulate the council official: ‘Come, let's establish a working group, let's do something’.”* [7S and 9S]

### Influential factors

The two municipalities with stronger declines in which the municipal board took a leading role differed with other municipalities in terms of municipal employees (i.e., enthusiastic, and consistently present council officials), resources (i.e., money, and time), and political support.

*A: “Just effort, people, council officials. Availability of money. Just time and hours. … yes, I [policy advisor] noticed this in everything. Also, they [municipality] really wanted to have a leading position.”* [8S]

*Q: “So they [municipality] reserved a budget on their own to…” A: “Yes, and in addition they had money that they isolated/extracted from the regional alcohol prevention project.”* [6S]

In addition, the role of the regional Public Health Service depended on input and allocation of resources to alcohol-related health problems within the organization and on the presence of experienced personnel.

*A: “I always say: ‘Person X [policy advisor of municipality 5S], you breath alcohol’. This person knows everything about alcohol and for ages this person’s focus is on alcohol.”* [1W]

Compared to this, in municipalities with weaker declines either money or time was limited or employees on certain positions in the municipal organization were constantly changing.

*A: “… during the year, budget cuts were necessary, there was a shortage in money, so the budget of public health was removed …”* [3W]

*A: “That is actually due to very practical reasons, namely time constraints of the council official that had to write this [implementation plan].*" [4W]

*A: “It is constantly someone else who sits over there [in the municipal board]. It is just very determinative … It all costs energy, ultimately, what [energy] you put in this you cannot put into the content.”* [1W]

## Discussion

### Main findings

Our study is one of the first that assessed how the development and implementation of integrated policies could have an impact on population health. We found that municipalities with stronger declines in youth alcohol consumption started to implement a package of more varied interventions, in which interventions with educational strategies were complemented with interventions with regulatory, enforcement, and media strategies. This more comprehensive package of interventions resulted from the involvement of sectors other than public health, and then especially the involvement of non-public health actors from outside the municipal organization. Their involvement was facilitated by framing youth alcohol consumption as a safety problem rather than a health problem. Local media played a key role in reframing this problem. The development and implementation of integrated alcohol policies was further facilitated by (a) participation in a regional alcohol prevention project, (b) leadership roles taken by either the municipality or by the regional Public Health Service, (c) sufficient resources, and (d) the expertise, enthusiasm, and continuity of key actors.

### Interpretations and implications

Our main finding suggests that stronger integrated alcohol policies, defined by the use of different types of intervention strategies and the involvement of different sectors, contributed to the reduction in youth alcohol consumption. In Noord-Brabant, until 2011, the development of integrated policies was at an early stage as different intervention strategies had yet to be developed and the emphasis was still on educational strategies in a school setting. In our previous study, we documented that most of the actions that were implemented in Noord-Brabant between 2007 and 2011 focused on adolescents under the age of 16 [19]. Moreover, between 2007 and 2011, not all municipalities involved different sectors from inside the municipal organization; collaboration between municipal policy sectors was limited. As large improvements can still be made, after 2011, most municipal organizations in The Netherlands invested in further development of integrated public health policies. New research should assess how much this further development had an impact on youth alcohol consumption, and what conditions were necessary to realize this impact. Our results implicate that regional collaboration can lead to integrated policies more easily than local collaboration. Further study in other national, regional and local contexts is needed to assess how integrated alcohol policies at higher administrative levels could foster the development and implementation of policies at lower levels.

A main question has been which factors could facilitate the development of integrated public health policies. We found that political agenda-setting was strongly supported by framing youth alcohol consumption as a societal problem rather than merely a public health problem. Some municipalities achieved this reframing with support of strong media attention. According to literature [20, 21], framing a problem and its consequences as transcending the public health field helps to alert different sectors of their responsibility to contribute to its solution. Framing is also important to move this solution from the political agenda towards its actual implementation [20]. According to policy theories [22, 23] and barrier models [24–26], political agenda-setting, and thus framing of a problem, depends on the needs and demands both in society and in politics.

Media contributes to the framing of a problem [27] and thus is important to influence political priority and agenda-setting [28]. In our study, we found that media attention could in addition be used during implementation of policies as a facilitative strategy, next to regulatory and enforcement strategies. As media is an important strategy to influence the public opinion (e.g., by raising awareness and priority) [20, 28], it could be used to increase the reach and uptake of interventions among citizens. Thus, for Noord-Brabant we conclude that media attention supported both the development of integrated policies and the implementation of specific alcohol prevention strategies.

Leadership is a facilitating factor for the development and implementation of integrated alcohol policies [9, 12, 14, 16]. We observed a stronger leadership, either from the municipal board (political leadership) or from the regional Public Health Service (program leadership), in municipalities with stronger declines in youth alcohol consumption. Leadership was found crucial to move from agenda-setting to the actual implementation of interventions. In our case study of Noord-Brabant, leadership came from established organizations that took the initiative to implement alcohol policies. Stronger leadership seemed to be related to more commitment, demonstrated by for example the enthusiasm of specific stakeholders. In other instances, leadership to health prevention projects could be organized differently, e.g. by involving policy entrepreneurs for health, health investment counselors, or health brokers [29]. Future research should evaluate different types of leadership for their impact on the development of integrated policies and, ultimately, population health.

We found that the implementation of integrated policies was facilitated by resources available within municipalities. Money and time were limited especially in municipalities with weaker declines in youth alcohol consumption. Previous studies also found that lack of money and time are main barriers for the implementation of integrated public health policies [12–15]. Their availability reflects the political priority that municipal boards give to integrated public health policies when deciding on the allocation of resources.

### Limitations

Our study has some limitations that need to be considered. First, we studied a sample of ten municipalities only. These municipalities represented a large contrast in trends in youth alcohol consumption within Noord-Brabant. Moreover, we observed considerable diversity between these municipalities in our detailed assessment of policy development and implementation. However, more variations could have been observed and stronger generalizations could have been made, if we were able to include more municipalities. For further research, we therefore recommend to apply our design of multiple case study to a large number of municipalities or other relevant constituencies.

Second, as some of the information was collected retrospectively, our results are subject to recall bias. For example, it is likely that we have underestimated the number and diversity of implemented interventions. This problem could have affected our findings if recall bias was different between municipalities with weaker and stronger declines in youth alcohol consumption. Such differences are however unlikely as at the interview start, advisors were unaware of whether their municipality had weaker or stronger declines. To avoid this potential problem in future studies, we recommend prospective multiple case studies, which have the additional advantage to measure the development and implementation of policies in greater detail than is possible retrospectively.

Third, we interviewed advisors from the regional Public Health Service, but no other officials involved in the policy process. Most of our interviewees had a detailed overview of the development and implementation of integrated policies, as they had been working for many years, mostly in an advising role, with their respective municipalities. Nevertheless, by interviewing additional stakeholders, such as aldermens, we would have obtained a more complete and perhaps more balanced view on the developments in each municipality.

## Conclusions

The results of this study suggest that integrated policies could have an impact on trends in youth alcohol consumption through a package of interventions that includes both media, regulatory, and enforcement strategies. The implementation of such a varied package requires the involvement of sectors other than public health, which can be facilitated by political priority and by framing public health problems as broader societal problems. Important to the success of some municipalities in Noord-Brabant between 2007 and 2011 were there participation in a regional alcohol prevention project, leadership, commitment and sufficient resources. Further application of our multiple case study approach is needed, not only to assess how much integrated policies could positively influence population health, but also to better understand under what conditions such impacts could be realized.
